# Huogu injection protects against SONFH by promoting osteogenic differentiation of BMSCs and preventing osteoblast apoptosis

**DOI:** 10.1007/s00441-023-03846-7

**Published:** 2023-12-02

**Authors:** Xin Zhang, Ziyu Li, Xilin Xu, Zhao Liu, Yuanyuan Hao, Fubiao Yang, Xiaodong Li, Ning Zhang, Yunlong Hou, Xiaofeng Zhang

**Affiliations:** 1 Luoyang Orthopedic-Traumatological Hospital of Henan Province (Henan Provincial Orthopedic Hospital), Luoyang, 471002 Henan China; 2 Graduate School, Heilongjiang University of Chinese Medicine, Harbin, 150000 Heilongjiang China; 3 The Third Affiliated Hospital of Heilongjiang University of Chinese Medicine, Harbin, 150000 Heilongjiang China; 4 The First Affiliated Hospital of Zhejiang University of Chinese Medicine, Hangzhou, 310000 Zhejiang China; 5 Shijiazhuang Yiling Pharmaceuticalco., ltd, Shijiazhuang, 050000 Hebei China; 6 Heilongjiang University of Chinese Medicine, Harbin, 150000 Heilongjiang China; 7 National Key Laboratory of Collateral Disease Research and Innovative Chinese Medicine, Shijiazhuang, 050000 Hebei China

**Keywords:** Huogu injection, BSMCs, Osteogenic differentiation, Osteoblasts, Apoptosis, SONFH

## Abstract

To investigate the effect and mechanism of Huogu injection (HG) on steroid-induced osteonecrosis of the femoral head (SONFH), we established a SONFH model in rabbits using horse serum and dexamethasone (DEX) and applied HG locally at the hip joint. We evaluated the therapeutic efficacy at 4 weeks using scanning electron microscopy (SEM), micro-CT, and qualitative histology including H&E, Masson’s trichrome, ALP, and TUNEL staining. In vitro, we induced osteogenic differentiation of bone marrow stromal cells (BMSCs) and performed analysis on days 14 and 21 of cell differentiation. The findings, in vivo, including SEM, micro-CT, and H&E staining, showed that HG significantly maintained bone quality and trabecular number. ALP staining indicated that HG promoted the proliferation of bone cells. Moreover, the results of Masson’s trichrome staining demonstrated the essential role of HG in collagen synthesis. Additionally, TUNEL staining revealed that HG reduced apoptosis. ALP and ARS staining in vitro confirmed that HG enhanced osteogenic differentiation and mineralization, consistent with the WB and qRT-PCR analysis. Furthermore, Annexin V-FITC/PI staining verified that HG inhibited osteoblast apoptosis, in agreement with the WB and qRT-PCR analyses. Furthermore, combined with the UPLC analysis, we found that naringin enhanced the osteogenic differentiation and accelerated the deposition of calcium phosphate. Salvianolic acid B protected osteoblasts derived from BMSCs against GCs-mediated apoptosis. Thus, this study not only reveals the mechanism of HG in promoting osteogenesis and anti-apoptosis of osteoblasts but also identifies the active-related components in HG, by which we provide the evidence for the application of HG in SONFH.

**Supplementary Information:**

The online version contains supplementary material available at 10.1007/s00441-023-03846-7.

## Introduction

Nontraumatic osteonecrosis of the femoral head (NONFH) is a common debilitating disease that causes loss of integrity of the subchondral bone structure and progresses to femoral head collapse (Agarwala and Vijayvargiya 2019). This disease frequently occurs in patients of any age (Zhao et al. 2015) and brings a heavy financial burden to the healthcare system worldwide. In the current clinical practices, conservative treatments (Wang and Wang 2019), such as physical therapy and pharmacotherapy, and tissue engineering treatments based on cytotherapy (Zhang et al. 2021) have been questioned for their efficiencies. The reality is, right now, more than 80% of NONFH cases finally develop into the late stage, and the prevalence of joint-replacing surgeries, including total hip arthroplasty (THA), increases with years (Takegami et al. 2016). In spite of the high surgery cost, THA has disadvantages such as the infection (Ahmed et al. 2019), dislocation (Petis et al. 2015), and longevity of implants (Peters et al. 2018). Thus, it is imperative to develop novel therapies for reconstitution of the osteonecrotic area in early stage NONFH.

Although the pathogenesis of NONFH has yet to be fully elucidated, many risk factors have been found to contribute to the progression of NONFH, among which steroids (glucocorticoids, GCs) abuse is the most commonly reported risk factor (Li et al. 2020) and increases the odds of NONFH by a factor of 35 (Zhao et al. 2015). Moreover, GCs excess could damage the self-renewal ability and attenuate osteogenic differentiation of bone marrow mesenchymal stem cells (BMSCs), resulting in a decrease of mature osteoblasts and disorder in the formation of new bone (Kang et al. 2016), thereby implicating BMSCs in SONFH progression. Over the recent years, accumulating evidence indicates that Wnt/β-catenin signaling pathway is a multicomponent cascade involving interaction of several proteins and is essential for proliferation, renewal, and differentiation of stem cells during embryonic development and adult tissue homeostasis, including the differentiation of MSCs into osteoprogenitor cells and chondrocytes (Han et al. 2020). However, in bone, overdose GCs have been reported to weaken Wnt signaling and osteoblastic differentiation of BMSCs, leading to decreased bone mass, ultimately resulting in the collapse of the femoral head (Clevers and Nusse 2012; Nusse and Clevers 2017; Houschyar et al. 2018).

In addition to bone loss, steroid-induced abnormal apoptosis is associated with SONFH. The inhibition of the proliferation and promotion of apoptosis of osteoblasts triggered by the excessive use of GCs, dominates in the pathogenesis of SONFH (Xu et al. 2021). GCs excess could cause a remarkable decrease in the expression of Bcl-2, an increase in cytochrome c (Cyt c) release, activation of caspase-3, and play a pivotal role in apoptosis associated with osteonecrosis of the femoral head (Yan et al. 2020). Among them, Bcl-2 is an anti-apoptotic protein that is localized to the outer mitochondrial membrane where it blocks the release of Cyt c from mitochondria by inhibiting apoptosis-induced mitochondrial pore formation (Mollazadeh et al. 2015; Tao et al. 2017). Furthermore, GCs-mediated upregulation of caspase-3 expression has been observed in SONFH patients (Xu et al. 2014) and functions as effector or “executioner” caspases, cleaving poly [ADP-ribose] polymerase (PARP) (Chen et al. 2018), that ultimately stimulates prominent apoptosis of osteocyte and osteoblasts and uniquely disrupts the new bone formation, resulting in low bone mineral density and collapse of the femoral head (Xu et al. 2021). In brief, SONFH arises from multiple risk factors; clinicians need to make allowances for these complex causes to prevent bone loss when treating SONFH.

Traditional Chinese formulae are under the guidance of the theory of traditional Chinese medicine, usually made up of several herbal medicines. The core principles of formulae are to find an interconnected, complementary, and interdependent relationship for each piece and combine them to yield more beneficial results in treating disease than in using them individually. To a certain extent, the aim of formulae could equate multiple-target actions. Huogu injection (HG) is mainly composed of DanShen (*Salvia miltiorrhiza Bge*), ChuanXiong (*Ligusticum chuanxiong hort*), and GuSuiBu (*Drynaria fortunei (Kunze) J.Sm*) and has been used in the second affiliated hospital of the Heilongjiang University of Chinese Medicine. HG plays the role of activating blood and qi, replenishing kidney, and strengthening bones according to the traditional Chinese medical theory, which could be translated to the understanding that the HG might improve the blood flow of the femoral head and reduce the bone loss. Although the possible mechanism of HG has not yet been thoroughly investigated, some herbs in this formula were proven to have the potential for SONFH.

Ultra-high performance liquid chromatography (UPLC) analysis shows there are 42 compounds. Among them, naringin (NG) is an active component extracted from GuSuiBu, and the previous paper have revealed that NG can promote proliferation and osteogenic differentiation of BMSCs via the Wnt/β-catenin signaling pathway (Zhang et al. 2009). Additionally, DanShen is currently used for the treatment of microcirculatory disturbance, which may improve blood circulation in SONFH (Huang et al. 2008). Salvianolic acid B (SB) extracted from DanShen has a protective effect by reducing the incidence of SONFH, effectively intervening in apoptosis through decreasing caspase-3 (Ma et al. 2020). Therefore, the present study utilized rabbit models of SONFH and attempted to investigate the possible underlying molecular mechanisms of HG.

In this present study, we aim to provide experimental evidence for the effects of HG on SONFH and to investigate the underlying mechanism to prove the multi-target therapeutics of HG. Our results proved that intracavitary injection of HG could promote osteogenesis by stimulating BMSCs osteogenic differentiation and inhibit apoptosis in the osteoblast differentiated from BMSCs. Given the HG-delivery way, substances could get access to lesions by which the osteogenic effect of NG and anti-apoptotic effect of SB was confirmed in vitro. Our data proved that HG would be a promising therapeutic option for SONFH.

## Methods and materials

### Animals and establishment of SONFH model

All experimental procedures and animal handling were performed with the approval of the Animal Care and Use Committee of Medical Research Institute of Yiling (NO. N2021021), in accordance with the National Institutes of Health Guide for the Care and Use of Laboratory Animals. Twenty healthy male New Zealand rabbits (1.8 ~ 2.3 kg, 5 ~ 6 months) and five male Sprague–Dawley rats (200 ~ 220 g, 2 ~ 3 weeks) were procured from Beijing Fulong Tengfei Experimental Animal Research Institute Co., Ltd. (Beijing, China) and housed in animal chambers maintained at 22 ± 2 °C with a relative humidity of 50 ± 5% and a 12/12-h light/dark cycle and provided with ad libitum food and water.

**Fig. 1:**
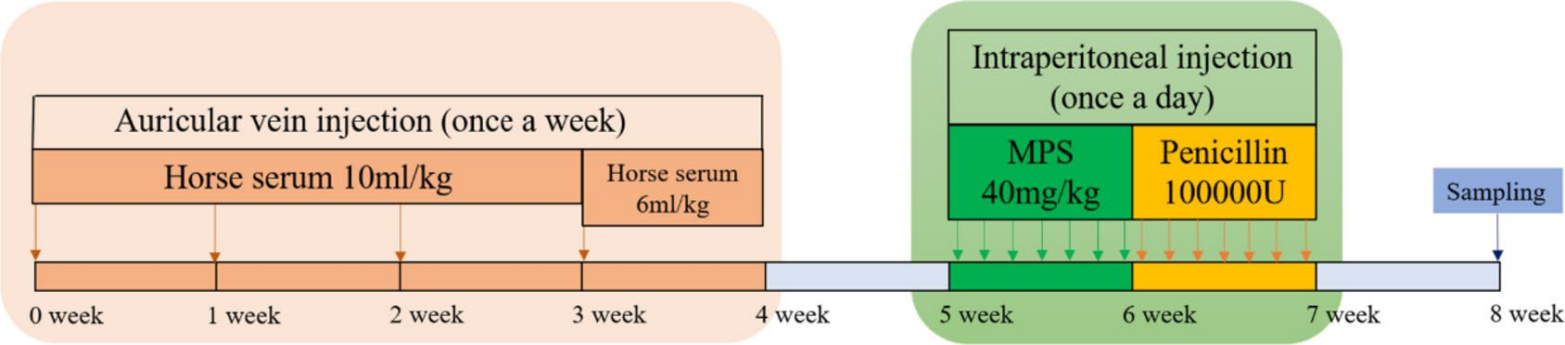
The protocols used to establish the SONFH models

The rabbits were randomly selected and divided into two groups: control group (*n* = 6) and pre-model group (*n* = 14). The method for replicating SONFH of rabbits was as follows (Fig. 1) (Cui et al. 2020): for the pre-model group, each experimental animal was injected with horse serum 10 ml/kg once a week through the ear vein. Three weeks later, the second horse serum (6 ml/kg) was given. After another 2 weeks, the methylprednisolone (MPS) was administered intraperitoneally with a dose of 40 mg/kg/d for 7 days. Subsequently, each rabbit was intraperitoneally administered with 100,000 U penicillin, once a day for 7 days, to prevent infection. For the control group, horse serum and MPS were replaced by an equal amount of saline. One week later, abnormal behavior was observed and 2 rabbits from pre-model groups were sacrificed randomly to confirm whether the SONFH models were successfully established by micro-CT. When the models were established successfully, the remaining rabbits in the pre-model group were randomly divided into model group (*n* = 6) and HG group (*n* = 6) for follow-up administration. For the HG group, the rabbits were given a HG of hip injection every third day for 4 weeks. The total dose of the double hips per rabbit was about 1 ml of HG (0.5 ml per hip joint). To control the variables, the control and model group were given an equal amount of saline hip injection.

### Micro-CT and scanning electron microscopy (SEM)

The left femurs from rabbits were dissected after the rabbits were euthanized and fixed for 48 h with 10% neutral-buffered formalin, then scanned and analyzed with a high-resolution micro-CT imaging system (Quantum GX2; PerkinElmer, USA). The scanner parameters were set at: X-ray kV: 50 kV, X-ray µA: 100 µA, scan time: 14 min, FOV: 6 mm. The morphologic changes of femoral heads were measured by micro-CT scanning. For the distal femur, the region of interest (ROI) selected for analysis was 5% of the femoral length from 1 mm above the growth plate to determine bone surface per bone volume (BS/BV), bone surface per total volume (BS/TV), bone volume fraction (BV/TV, %), bone mineral density (BMD), trabecular number (Tb.N, 1 mm^−1^), trabecular thickness (Tb.Th, mm), trabecular separation (Tb.Sp, mm), and connectivity density (Conn.D, 1 mm^−3^). For cortical bone, data include total bone area (Tt.Ar), cortical area (Ct.Ar), and cortical thickness (Ct.Th, mm).

Tissues of rabbit femoral heads for electron microscopic examination were fixed with 2.5% glutaraldehyde, and 2.0% paraformaldehyde in 0.1M sodium cacodylate buffer, pH7.4, overnight at 4 °C. After subsequent buffer washes, the samples were post-fixed in 2.0% osmium tetroxide for 1 h at room temperature and then washed with buffer followed by distilled water. After dehydration through a graded ethanol series, the tissue was infiltrated. Finally, morphologies of rabbit femoral heads were observed with SEM (Hitachi S-3000N, Japan) at 15 kV, after gold coating.

### Histological and TUNEL staining

Femoral-head samples went through fixation for 48 h in 10% formalin and decalcification for 4 weeks in 10% ethylenediaminetetraacetic acid (EDTA, Sigma, USA). Then, specimens were decalcified and embedded in paraffin and cut into 5 µm thick sections. H&E, Masson’s trichrome (Solarbio, G1340, China), and ALP staining (Solarbio, G1480, China) were performed subsequently according to the manufacturer’s protocol, and finally an examination under a microscopic light by using a scan scope digital slide scanner (Hamamatsu, Japan). H&E staining was used to observe the detailed view of specimens and to evaluate the trabecular structure. Masson’s trichrome staining was employed to determine the growth plate thickness of sections. An alkaline phosphatase (ALP) staining was performed to calculate the number of osteocytes.

For TUNEL staining, bone sections of rabbits were treated and stained with a TUNEL reaction mixture following the manufacturer’s instructions (Wanleibio, WLA127a, China). Nuclei were co-stained for 5 min with DAPI solution (Alading, D106471-5 mg, China), and images were captured under a microscope (U-HGLGPS, Olympus, BX53, Japan). And the apoptosis rate was calculated using Image J (version 1.8.0, open source).

### UPLC-Q-TOF/MS was used to identify the active components of HG

The HG has been used in the second affiliated hospital of the Heilongjiang University of Chinese Medicine. UPLC-Q-TOF/MS system (Manchester, UK) was used to identify the active components of HG as described in the previous paper (Yang et al. 2022). Mass spectra were acquired in both negative and positive modes, and non-target compound identification was further conducted based on obtained fingerprints, and the compound was finally confirmed by the comparison with the authentic compound.

### Isolation, culture, and characterization of BMSCs

BMSCs were isolated from bilateral femurs and tibia of rats, as described previously (Yang et al. 2022). The BMSCs were cultured in MEM-α (Gibco, 12,571–063, USA) supplemented with 10% FBS (Gibco, 12,664–025, USA), 1% penicillin and streptomycin (Gibco, 15,140–122, USA), and maintained at 37 °C with 5% CO_2_. For cell surface marker characterization using flow-cytometry, 1 × 10^6^ cells at passage 3 were stained with 1:100 dilution of fluorescein isothiocyanate3-conjugated anti-CD29 (Abcam, ab27947, UK), CD45 (Abcam, ab10558, UK), and CD90 (Abcam, ab226, UK) antibodies, respectively. Corresponding isotype-matched control antibodies were used for negative controls. Samples were analyzed using the BD FACSCanto™ II Flow Cytometer (BD Biosciences).

### Cell viability

The cell viability was detected using MTS regents (Promega, G3581, USA) according to the manufacturer’s recommendations. The cells were seeded in a 96-well plate at a density of 1 × 10^5^ cells/well and then incubated at 37 °C and 5% CO_2_. MTS solution was added to each well and incubated for 3 h. Then, the absorbance was detected at 490 nm and 630 nm (reference) with a Microplate Reader (Infinite^®^ M200 PRO, Tecan, Switzerland) to determine the OD value of each well.

### Osteogenic induction of BMSCs and medication administration

For osteogenic differentiation, BMSCs were seeded in a 6-well culture plate at a density of 1 × 10^5^ cells/well with the osteogenic-induced medium (OIM), which is a complete medium supplemented with 10 nM dexamethasone (MCE, HY-14648, USA), 10 mM β-glycerophosphate (Sigma, A5422-25G, USA), and 0.05 mM ascorbic acid-2-phosphate (Sigma, A4544-25G, USA). Subsequently, the cells were cultured and the medium was changed every 3 days. To detect the effects of HG and NG on the osteogenic differentiation of BMSCs, the medication treatments in different groups were as follows: control group: OIM; model group: OIM + 10^−6^ mol/l DEX; HG group: OIM + 10^−6^ mol/l DEX + 15 µl/ml HG; NG group: OIM + 10^−6^ mol/l DEX + 100 µM NG. In addition, in order to detect the effects of HG and SB on osteoblasts apoptosis, the BMSCs were cultured in OIM for 14 days to obtain osteoblasts as described in the previous paper (Tang et al. 2018). Then the osteoblasts administration was conducted as follows: control group: OIM; model group: OIM + 10^−6^ mol/l DEX; HG group: OIM + 10^−6^ mol/l DEX + 15 µl/ml HG; SB group: OIM + 10^−6^ mol/l DEX + 100 µM SB.

### ALP staining and Alizarin red staining (ARS)

The osteogenic differentiation of the cells was measured using ALP and ARS as early and late osteogenic markers, respectively (Golafshan et al. 2020). On the 14th day of osteogenic differentiation, OIM was discarded and ALP staining (Solarbio, G1480, China) was performed to detect ALP activity, following the manufacturer’s instructions. Briefly, cells in 6-well plate were gently washed with PBS solution by three times, then the cells were fixed with ALP fixative solution for 3 min at room temperature. After washing, the cells were rinsed in ALP incubation buffer for 20 min, protected from light. After that, distilled water was used to wash the samples again; counterstaining was performed by incubating the cells in a nuclear fast red solution for 5 min. Finally, samples were washed again and photographed under the standard light microscopy (Olympus, BX53, Japan), and ALP activity was quantified with Image J (version 1.8.0, open source); values are expressed as the percentage of the area measured.

After 21 days of osteogenic induction, the cells in 6-well plate were stained with Alizarin Red (Solarbio, G1450-100, China) to assess the mineralization according to the manufacturer’s instructions. In short, the cells in 6-well plate were fixed with 4% paraformaldehyde for 15 min, washed with PBS three times, and stained with Alizarin Red S solution for 30 min. After washing with distilled water, the stained cells were observed and photographed under the microscope (Olympus, BX53, Japan), and the mineralization was quantified with Image J (version 1.8.0, open source); values are expressed as the percentage of the area measured.

### Assay of cell apoptosis

The annexin V-FITC/PI apoptosis assay was used to quantify osteoblasts apoptosis in vitro. The osteoblasts derived from BMSCs were seeded in 12-well culture plates with the cell concentration of 2 × 10^5^/well and cultured in the presence of OIM, DEX (10^−6^ mol/L), HG (15 µl/ml) or SB (100 µM) for 72 h. At the end of culture, cells were washed twice with cold PBS, followed by resuspension in 100 µl binding buffer. Next, 5 µl Annexin V-FITC and 10 µl PI were used for 15 min of staining with 50 µg/ml RNase A (Sigma-Aldrich, USA), followed by 1 h of cell incubation in an incubator in the dark. A flow cytometer (Becton, USA) was used for cell assay. FlowJo software (Ashland, USA) was utilized for data analysis.

### Western blotting assay

Cells were washed with 1 × PBS and lysed in RIPA buffer (Servicebio, G2002, China) supplemented with protease inhibitor cocktail and PMSF (Servicebio, G2008, China) on ice for 30 min. The cell lysates were subsequently centrifuged at 14,000 g at 4 °C for 15 min. The samples were heated at 95 °C for 5 min in a sample buffer containing 2% SDS and 1% 2-mercaptoethanol; lysates were separated using 10–12% SDS-PAGE and electro-transferred onto a polyvinylidene fluoride membrane (PVDF, Millipore, USA). The PVDF with proteins was then blocked with 5% (w/v) skimmed milk for 1.5 h to block the non-specific sites on blots. The primary antibodies (listed in Supplementary data 1) dissolved in the blocking buffer were used to determine the targeting protein blots overnight at 4 °C. After adding the anti-rabbit or anti-mouse secondary antibody (listed in Supplemental data 1) for 1 h at 37 °C, the protein bands on the membranes were detected with the Odyssey infrared imaging system (LI-COR^®^ Biosciences, USA) and analyzed as specified in the Odyssey software manual. β-actin was used as the internal reference.

### Total RNA extraction and quantitative reverse transcription-polymerase chain reaction (qRT-PCR)

The total RNA of cells was extracted using an RNA extraction kit (Promega, LS1040, USA) according to the manufacturer’s instruction. The RNA concentration was measured with a NanoDrop One instrument (Thermo Fisher Scientific, USA). The cDNA was prepared using the Eastep™ RT Master Mix (5X) (Promega, LS2050, USA). qRT-PCR was performed using GoTaq^®^ qPCR Master Mix (Promega, A6001, USA) in T100TM Thermal Cycler System (BioRad, USA). Relative gene expression was normalized by GAPDH for osteogenesis and apoptosis using a 2^−ΔΔCt^ method. The primers are listed in Supplemental data 2.

### Statistical analysis

Experimental results are presented as mean ± standard deviation (*n* = 3) for each group. Statistical analysis was performed in GraphPad Prism 9. The data were analyzed using two-tailed Student’s *t*-test between two groups and one-way analysis of variance followed by Dunnett’s post hoc tests when groups were more than two. “ns” indicated *P* ≥ *0.05*, *P* < *0.05* suggested a statistical difference, and *P* < *0.01* considered statistically significant.

## Results

### HG alleviated GC-induced bone destruction and bone loss in rabbits

**Fig. 2:**
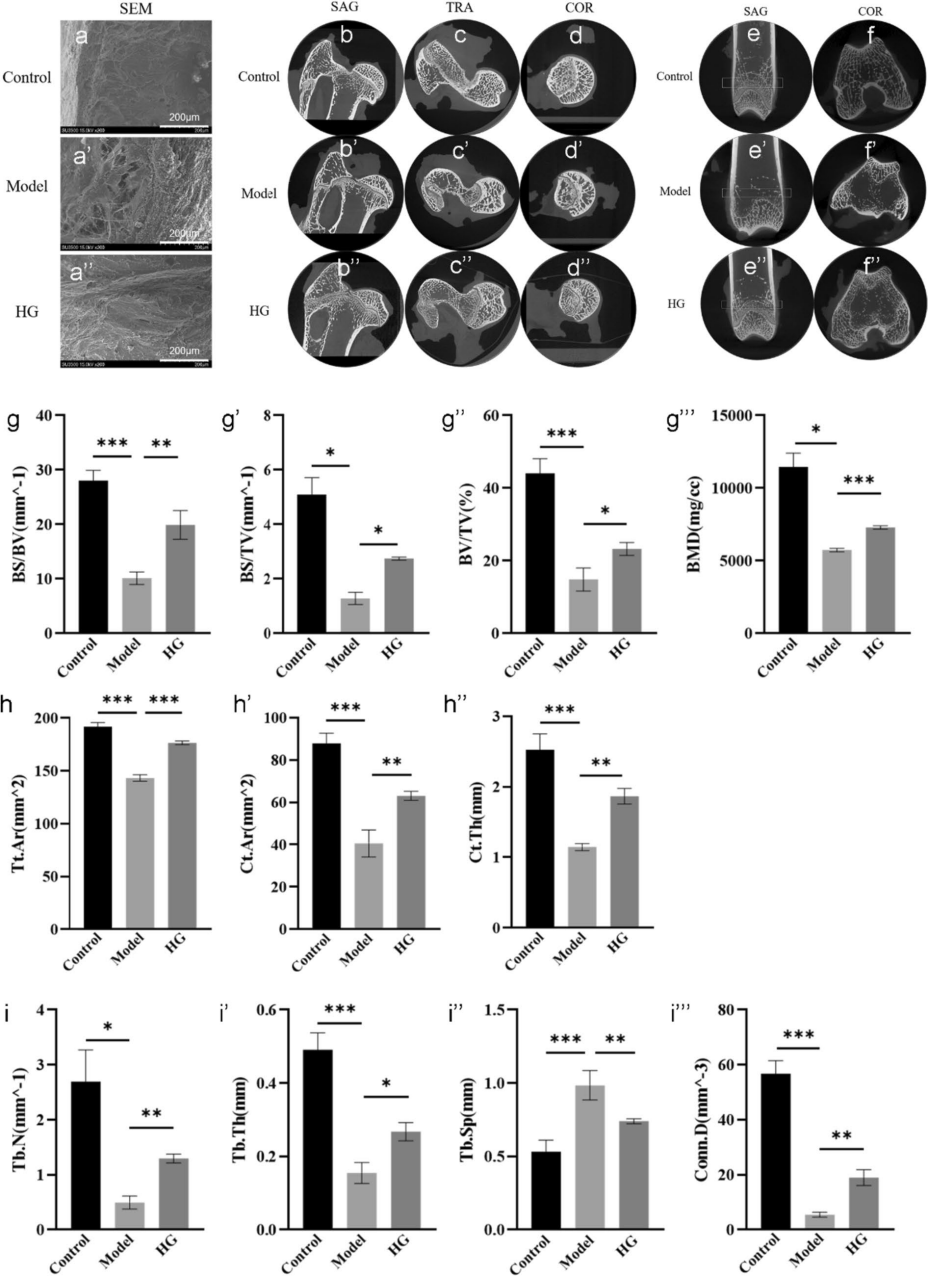
Analysis of the rabbit bone structure and microstructural parameters by SEM and Micro-CT. **a**–**a’’** SEM images of rabbit femoral heads. **b–d’’** Sagittal, transverse, and coronal micro-CT images of rabbit femoral heads. **e–f’’** Sagittal and coronal micro-CT images of the rabbit distal femurs. **g–i’’’’** The bone parameters of the rabbit distal femurs analyzed by micro-CT. ^*^P < 0.05, ^**^P < 0.01, ^***^P < *0.001*

As presented in Fig. 2, the results of SEM (Fig. 2a–a’’) and micro-CT (Fig. 2b–d’’) illustrated bony destruction and multiple cavities in the femoral head, trabecular bone dispersion, and even fracture in the model group induced by DEX. Meanwhile, the images of the rabbit distal femur in Fig. 2e–f’’ demonstrated that the trabecular bone in the model group was sparse, narrowed, and fractured. Whereas in the HG group, those severe signs of the rabbit femoral head and distal femur were significantly alleviated (Fig. 2a–f’’). In addition, the bone microstructural parameters of the rabbit distal femur were analyzed by micro-CT. And the results suggested that, in the HG group, BS/BV, BS/TV, BV/TV, BMD, Tb.N, Tb.Th, Conn.D, Tt.Ar, Ct.Ar, and Ct.Th were significantly increased (*P* < *0.05*), whereas Tb.Sp was significantly decreased compared with the model group (*P* < *0.01*) (Fig. 2g–i’’’’), indicating that significant poor bone quality caused by DEX was obviously ameliorated after HG administration. In conclusion, HG could significantly alleviate GCs-induced bone injury and has a positive effect on the treatment of SONFH.

### HG not only stimulated bone formation but also inhibited the apoptosis induced by GCs

**Fig. 3:**
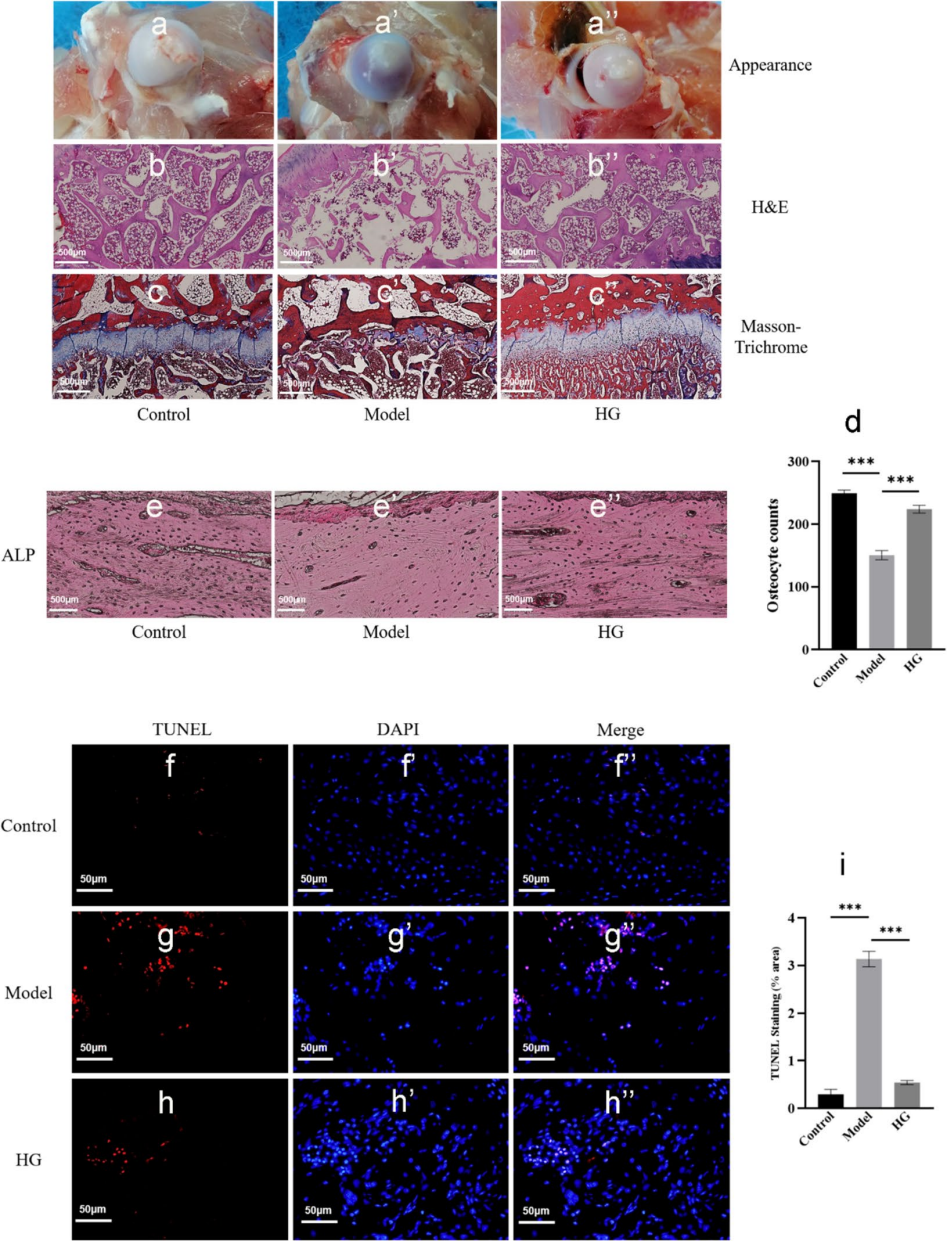
The histological and TUNEL analysis of GC-induced ONFH in rabbits. **a**–**c’’** The appearance and histological analysis of rabbit femoral heads. **d**–**e’’** Quantitative analysis of osteocytes by ALP staining. **f**–**i** Quantitative analysis of TUNEL staining. ^*^P < 0.05, ^**^P < 0.01, ^***^P < *0.001*

As displayed in Fig. 3a–a’’, the femoral head had a white alabaster appearance and an even surface in the control group. Conversely, an evident cyanotic surface of the femoral head was observed in the model group, which indicated that there is hemorrhage and necrosis of the femoral head (Lin et al. 2019). However, the femoral head in the HG group exhibited an intermediate appearance compared with the control and model group, and the results of H&E staining (Fig. 3b–b’’) verified the bony devastation, multiple cavities of the femoral head, and reductions in trabecular thickness and number in the model group induced by GCs. Whereas, in the HG group, the lesions mentioned above markedly ameliorated (Fig. 3b–b’’). And the results revealed that HG substantially preserved bone quality and trabecular number compared with the model group, which was consistent with the micro-CT and SEM results. Additionally, the results of Masson’s trichrome staining (Fig. 3c–c’’) manifested that the content of bone collagen increased considerably in the HG group, indicating that HG is crucial for collagen synthesis, and, consequently, for osteogenesis (Kuznetsov et al. 2013).

Furthermore, the ALP staining analysis (Fig. 3d–e’’) illustrated that osteocyte numbers in the HG group were increased compared with the model group (*P* < *0.001*), implying that HG contributed to the increased bone cells. Additionally, TUNEL staining demonstrated that the positive number of TUNEL staining cells in the model group were significantly upregulated compared with the control group (Fig. 3f–i). However, compared with the model group, the number of TUNEL-positive-stained cells was substantially less in the HG group (*P* < *0.001*) (Fig. 3f–i). Thus, these findings revealed that the upregulation of apoptosis induced by GCs in the model group can be downregulated by HG.

In conclusion, the results manifested that HG contributed to the increased bone formation and decreased apoptosis, by which we provided the experimental evidence in vivo that HG is able to improve the SONFH.

### Chemical composition analysis of HG

**Fig. 4:**
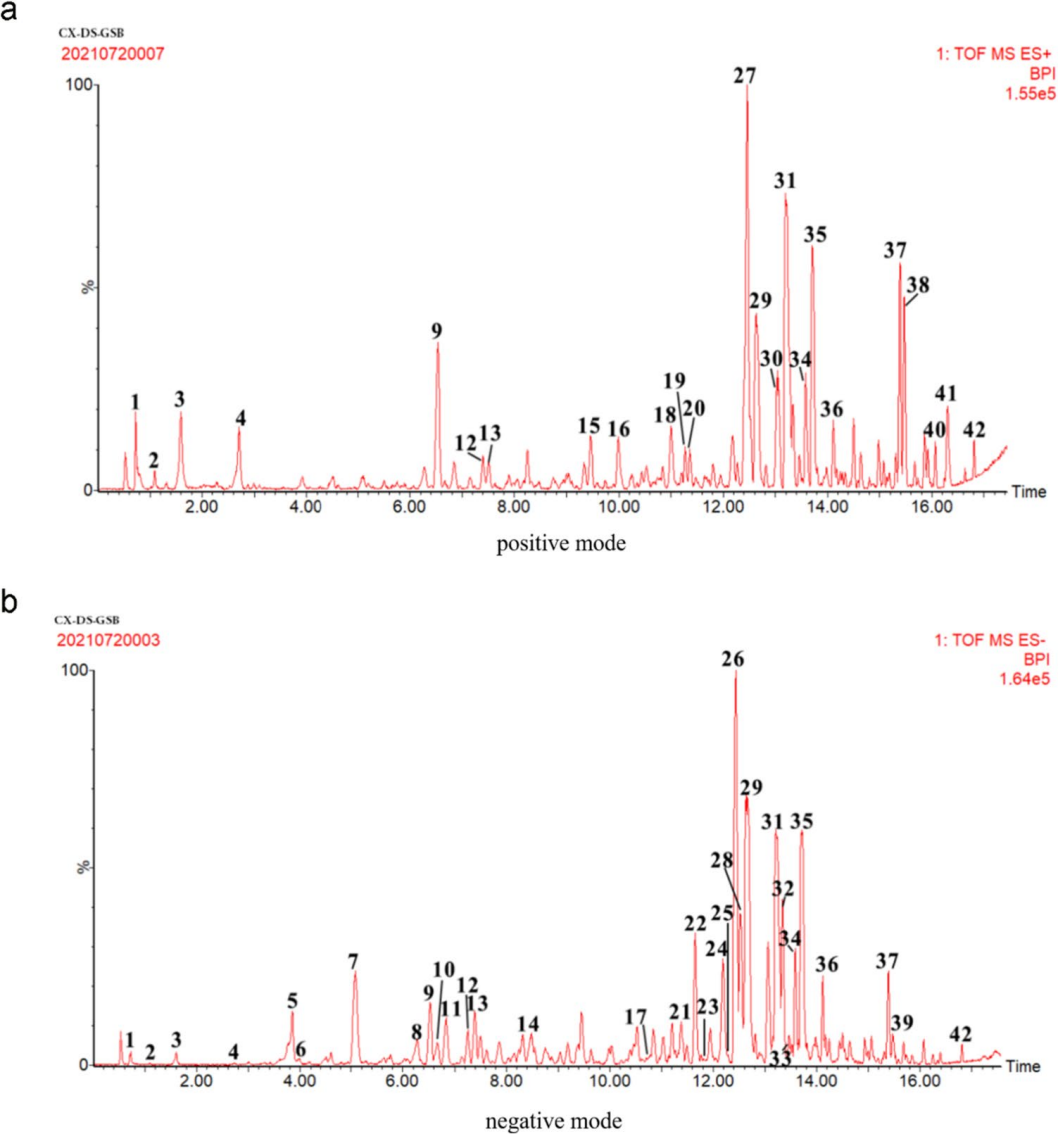
UPLC-Q-TOF–MS/MS chromatographic fingerprinting of HG

Previous experiments have demonstrated the promotion on bone formation and anti-apoptosis of HG in vivo. Indeed, HG, a compound Chinese medicine containing multiple components, is worthy of further study and the effective constituents should be identified. Therefore, the chemical composition of HG was evaluated using positive and negative ion mode UPLC-Q-TOF–MS (Fig. 4) as described in the previous paper (Yang et al. 2022) (presented at Supplemental data 3). The results revealed that NG and SB were identified as the active ingredients of HG. Existing research results have illustrated that NG promotes BMSCs differentiation towards an osteogenic fate via the Wnt/β-catenin signaling pathway (Saud et al. 2019), and SB has anti-apoptotic activities through inhibition of caspase-3 pathways (Chen et al. 2017). Thus, the effects of NG and SB were ulteriorly examined in subsequent experiments, providing a theoretical basis for elucidating the mechanism underlying the effects of HG on SONFH.

### Characterization of rat BMSCs and detecting the optimal concentration of HG

**Fig. 5:**
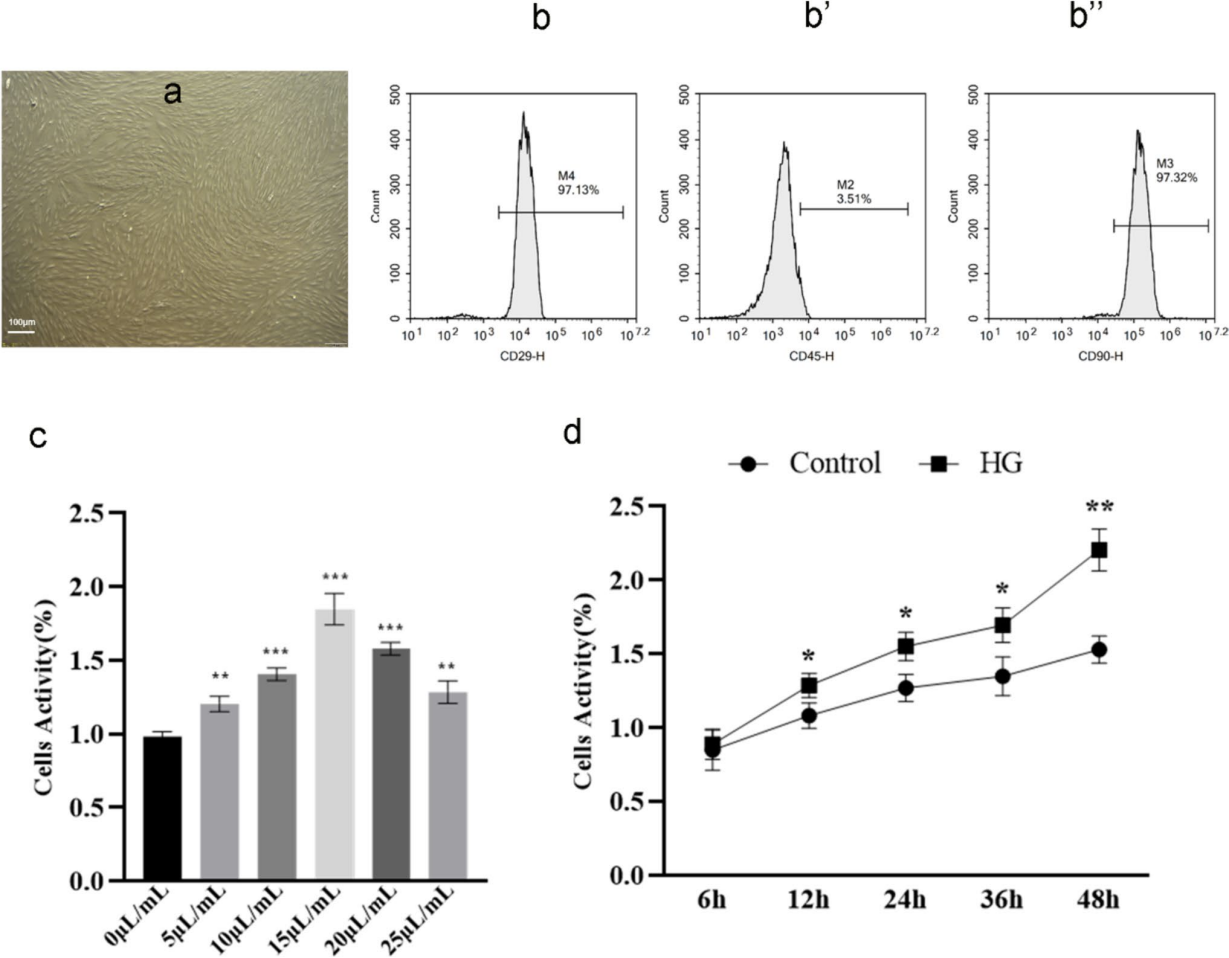
Characterization of rat BMSCs and detecting the optimal concentration of HG. **a** Morphological features of BMSCs. **b**–**b’’** Flow cytometry analysis of BMSCs surface markers. **c** HG affects cell proliferation in a dose-dependent manner. **d** The effect of 15 µl/ml concentration of HG on cell proliferation at different time points. ^*^P < 0.05, ^**^P < 0.01, ^***^P < *0.001*

As described in our previous paper (Yang et al. 2022), BMSCs from rat bone marrow showed a fibroblast-like shape and whirlpool-like distribution (Fig. 5a). For cell surface marker characterization, the results of flow cytometry analysis manifested that the primary BMSCs belonged to a population of nonhematopoietic, nonendothelial but mesenchyme-derived stem cells (Fig. 5b–b’’).

In addition, we set the different concentration gradients of HG to determine the optimal concentration by MTS assay, and it was confirmed by cell viability experiment that HG with 15 µl/ml obviously promoted the proliferation of rat BMSCs in a time-dependent manner (Fig. 5c, d). Therefore, we selected the 15 µl/ml HG for subsequent experiments in vitro.

### HG promoted osteogenic differentiation and matrix mineralization of BMSCs

**Fig. 6:**
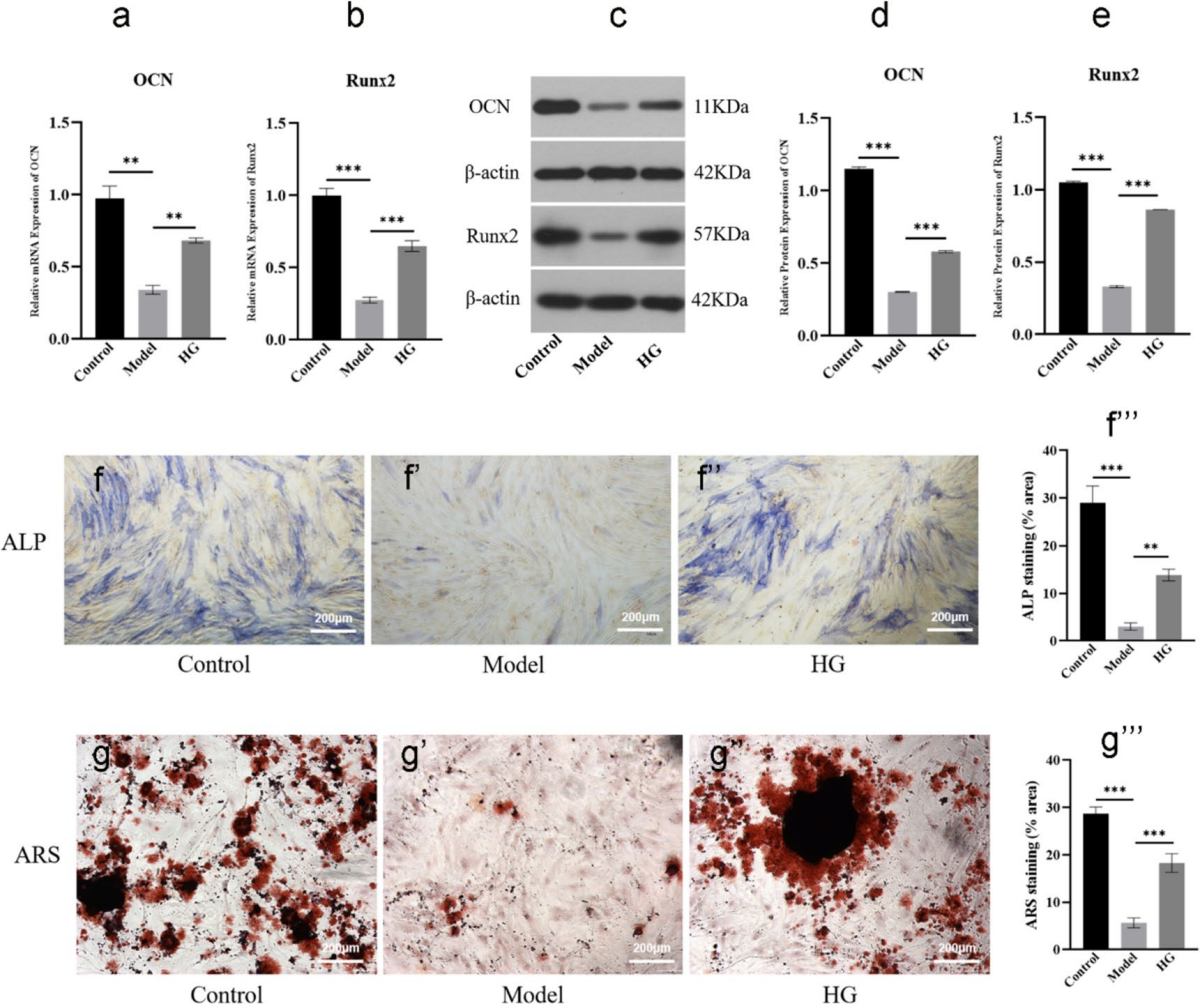
HG promoted osteogenic differentiation and matrix mineralization of BMSCs. **a**, **b** qRT-PCR analysis of the mRNA expression of OCN and Runx2 in different groups. **c**, **d**, **e** Western blot analysis of the protein expression of OCN and Runx2 in different groups. **f**–**f’’’** Representative images and quantitative analysis of ALP staining. **g**–**g’’’** Representative images and quantitative analysis of ARS. ^*^P < 0.05, ^**^P < 0.01, ^***^P < *0.001*

In order to study the role of HG in vitro, we performed western blot and qRT-PCR analysis to study the changes of osteogenic differentiation-related markers. The results demonstrated that HG significantly promoted osteogenic differentiation-related biomarkers, such as osteocalcin (OCN) and runt-related transcription factor 2 (Runx2) (Lee et al. 2014; Komori 2019), increased at mRNA and protein expression levels (*P* < *0.01*) (Fig. 6a–e), suggesting its promotion of osteogenic differentiation of BMSCs. Furthermore, ALP staining and ARS were performed to determine osteogenic differentiation and matrix mineralization. The results of ALP analysis indicated that the downregulated ALP expression caused by DEX in model group was upregulated by HG (*P* < *0.01*) (Fig. 6f–f’’’), and the ARS results also supported the salvage effect of HG treatment on osteogenesis from the perspective of mineralization (*P* < *0.001*) (Fig. 6g–g’’’). Taken together, these results suggested that HG promoted BMSCs osteoblastic differentiation, indicating its potential as a valid drug in the treatment of SONFH.

### NG stimulated osteogenic differentiation of BMSCs via the Wnt/β-catenin pathway

**Fig. 7:**
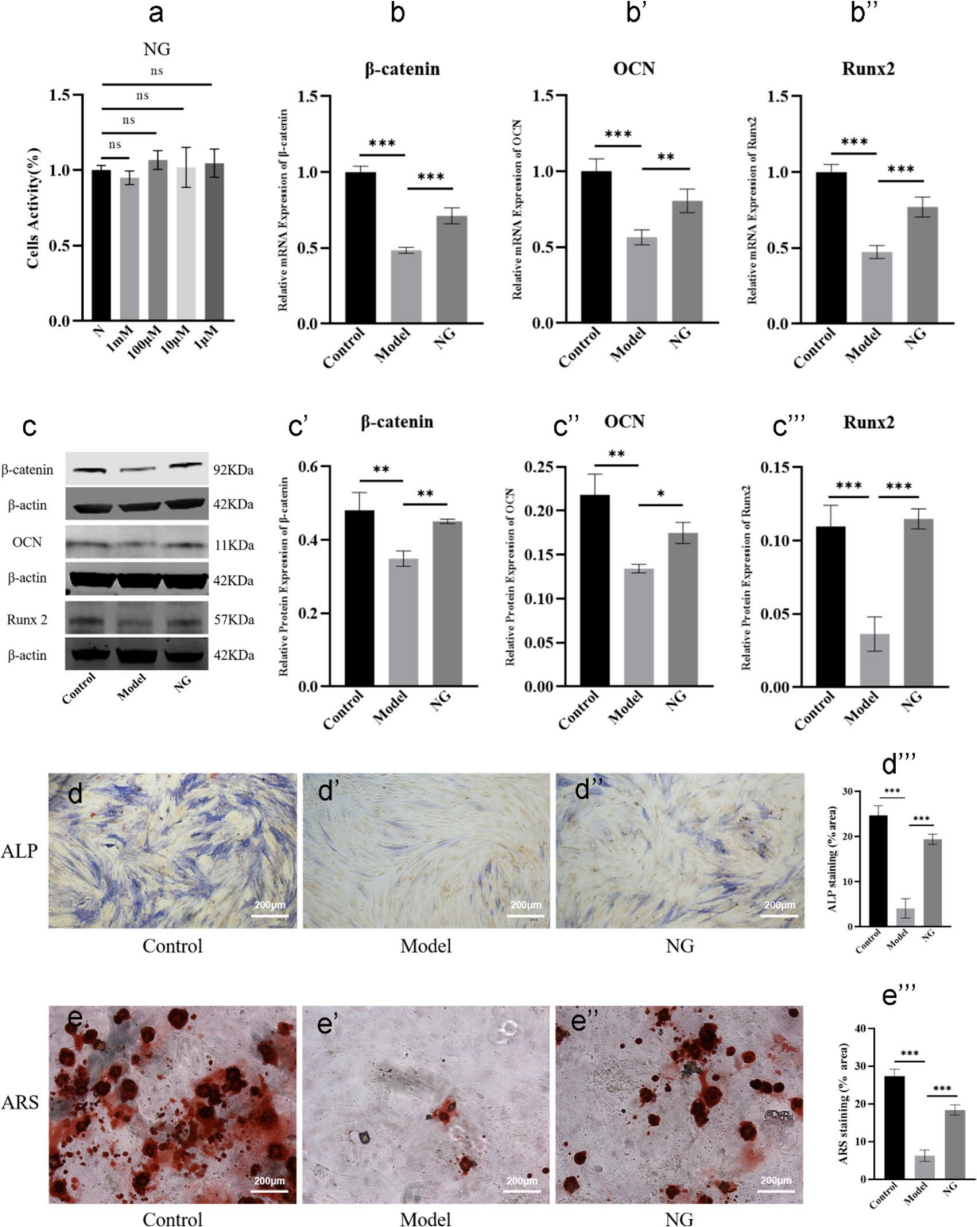
NG stimulated osteogenic differentiation and matrix mineralization of BMSCs. **a** Drug concentration detecting of NG. **b**–**b’’** qRT-PCR analysis of the mRNA expression of β-catenin, OCN, and Runx2 in different groups. **c**–**c’’’** Western blot analysis of the protein expression of β-catenin, OCN, and Runx2 in different groups. **d**–**d’’’** Representative images and quantitative analysis of ALP staining. **e**–**e’’’** Representative images and quantitative analysis of ARS. “ns” indicates *P* ≥ 0.05, ^*^P < 0.05, ^**^P < 0.01, ^***^P < *0.001*

Combined with the UPLC analysis in Supplemental data 3, NG was detected as the active ingredient of HG. Prior to researching the signaling pathways involved in NG-mediated enhancement of osteogenic differentiation, the optimal concentration of NG was determined. As displayed in Fig. 7a, at the concentration of 1 mM, 100 µM, 10 µM, 1 µM NG after 24 h, we did not see a substantial difference between the normal and the administration group (*P* > *0.05*). For the proper dosing and cost effectiveness, we investigated the effects of NG at the concentration of 100 µM in the following experiment.

The Wnt/β-catenin pathway has been revealed to be intimately involved in the osteogenic differentiation of BMSCs (He et al. 2019). Thus, we researched the effects of NG on the expression of genes (β-catenin, Runx2, and OCN) involved in osteogenesis of BMSCs (He et al. 2019). qRT-PCR and western blot assays demonstrated that, compared with the model group, 100 µM NG treatment for 14 days generated a considerable increase in the mRNA and protein levels for β-catenin, Runx2, and OCN (*P* < *0.05*) (Fig. 7b, c’’’). In addition, with the administration of NG (100 µM), a substantial elevation in the ALP expression was observed after 14 days compared with the model group (*P* < *0.001*) (Fig. 7d–d’’’). Next, BMSCs osteogenic differentiation was determined by ARS after cultured in OIM for 21 days. With the administration of NG (100 µM), the formation of calcium nodules was found to be increased, particularly significant with the treatment of DEX in the model group (*P* < *0.001*) (Fig. 7e–e’’’). In conclusion, we confirmed that NG enhanced osteogenesis of BMSCs by upregulating β-catenin expression via the Wnt/β-catenin pathway.

### HG attenuated apoptosis of osteoblasts derived from BMSCs in vitro

**Fig. 8:**
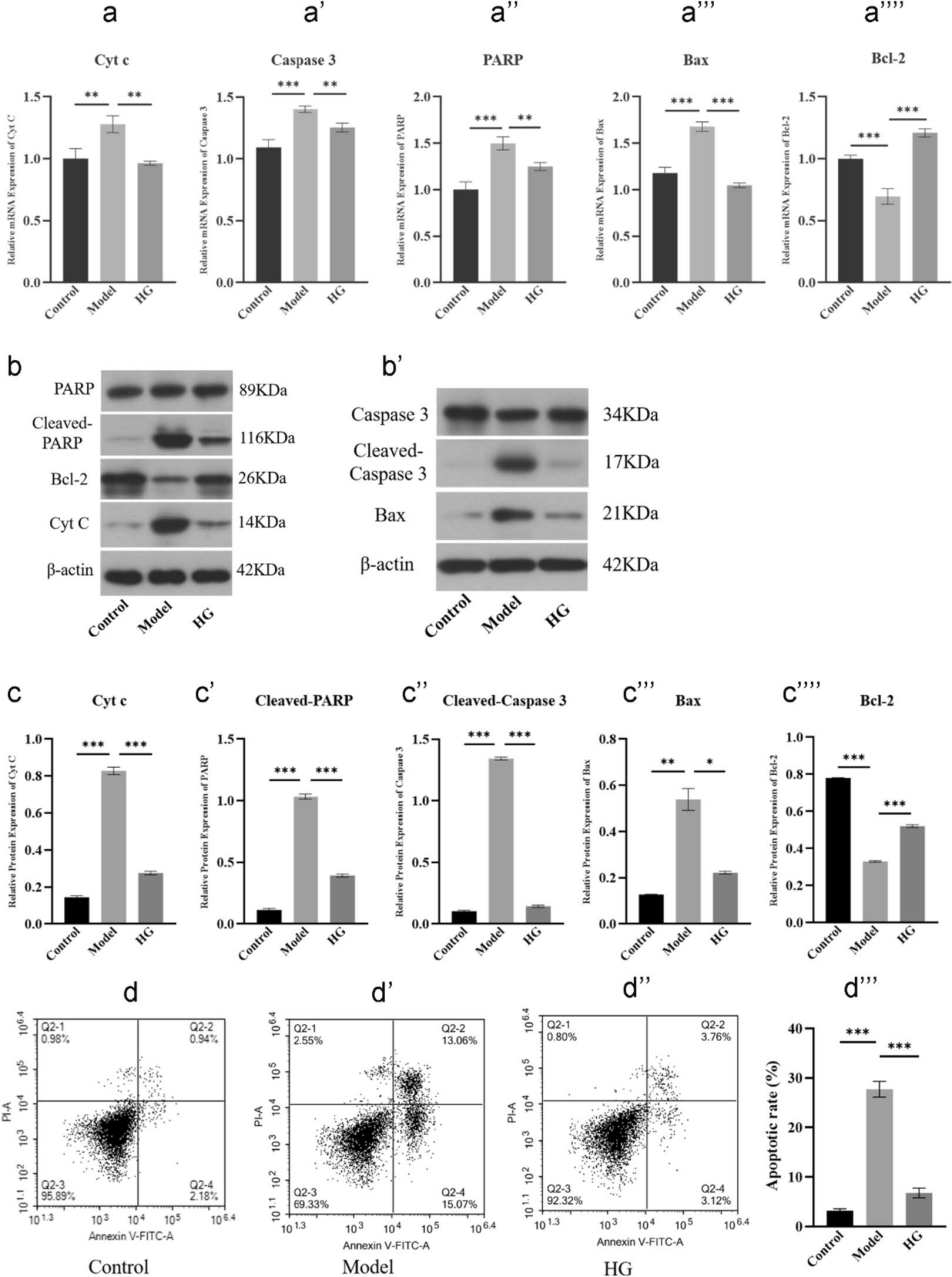
HG inhibited apoptosis of osteoblasts in vitro. **a**–**a’’’’** qRT-PCR analyses of the mRNA expression of Cyt c, PARP, caspase-3, Bax, and Bcl-2 in different groups. **b**–**c’’’’** Western blot analysis of the protein expression of Cyt c, PARP, caspase-3, Bax, and Bcl-2 in different groups. **d**–**d’’’** Evaluation of the apoptosis rate in different groups using Annexin V-FITC/PI staining. ^*^P < 0.05, ^**^P < 0.01, ^***^P < *0.001*

In order to research the role of HG in anti-apoptosis of osteoblasts derived from BMSCs, we performed western blot and qRT-PCR analyses to research the changes of apoptosis related markers. These results proved that HG significantly inhibited the expression of proapoptotic members, such as Cyt c, cleaved-PARP, cleaved caspase3, and Bax at the mRNA and protein levels (*P* < *0.01*), but promoted the mRNA and protein expression of Bcl-2, a member of antiapoptotic family (Mollazadeh et al. 2015) (*P* < *0.001*) (Fig. 8a–c’’’’), indicating its antiapoptotic effect on osteoblasts. Furthermore, the results of Annexin V-FITC/PI staining analysis manifested that DEX administration fostered apoptosis of osteoblasts derived from BMSCs in model group. Intriguingly, HG administration visibly decreased the apoptosis rate of osteoblasts compared with the model group induced by DEX (*P* < *0.01*) (Fig. 8d–d’’’). Taken together, the results indicated that HG attenuated the negative effect of GCs on osteoblasts apoptosis, implying its potential as an effective anti-apoptosis drug in the treatment of SONFH.

### SB inhibited apoptosis of osteoblasts derived from BMSCs via the caspase-3 pathway

**Fig. 9:**
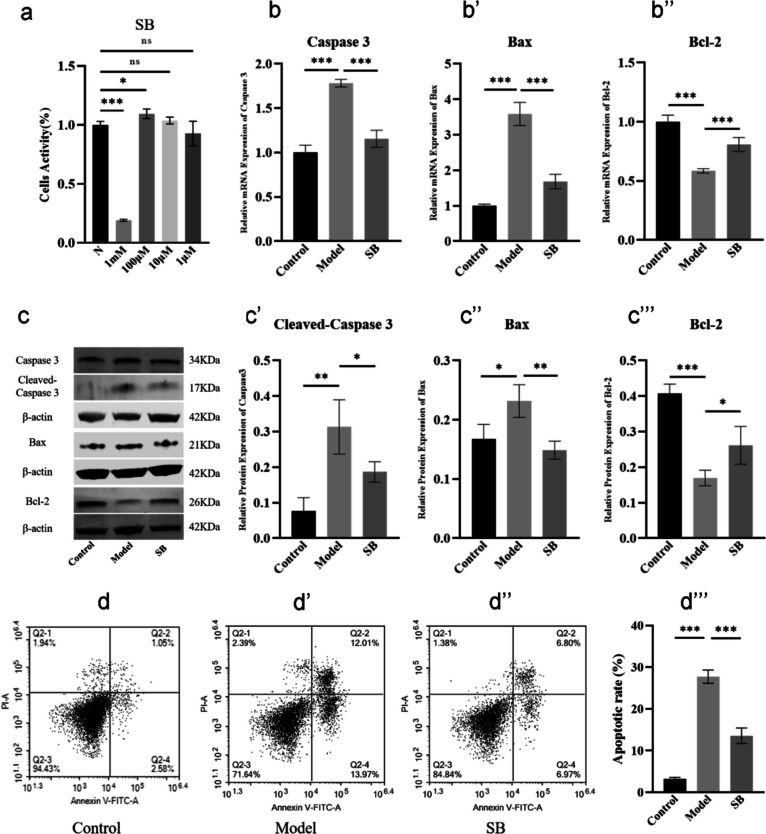
SB inhibited osteoblasts apoptosis via the caspase-3 pathway. **a** Drug concentration detecting of SB. **b**–**b’’** qRT-PCR analyses of the mRNA expression of caspase-3, Bax, and Bcl-2 in different groups. **c**–**c’’’** Western blot analysis of the protein expression of caspase-3, Bax, and Bcl-2 in different groups. **d**–**d’’’** Evaluation of the apoptosis rate in different groups using Annexin V-FITC/PI staining. “ns” indicates *P* ≥ 0.05, ^*^P < 0.05, ^**^P < 0.01, ^***^P < *0.001*

Combined with the UPLC analysis in Supplemental data 3, SB was identified as the active compound of HG. As shown in Fig. 9a, at the concentration of 100 µM, 10 µM, and 1 µM SB after 24 h, we did not see a significant difference between the normal and the administration group (*P* > *0.05*). When SB concentration was increased to 1 mM, the difference between the normal and the administration group was significant (*P* < *0.001*), with an over 80% decrease in the cell activity rate for the cells treated with SB. Therefore, we investigated the therapeutic effects of SB at the concentration of 100 µM in the following experiment.

DEX can induce apoptosis in osteoblasts (Xu et al. 2021), on the other hand, to examine the apoptotic signaling during DEX-induced osteoblasts apoptosis, we examined the expression of caspase-3 and Bax of which the apoptosis-related protein, and Bcl-2, an inhibitory protein for apoptosis (He et al. 2019). qRT-PCR and western blot results indicated that SB obviously suppressed the DEX-induced expression of apoptosis-related proteins caspase-3 and Bax (*P* < *0.0*5), it also prevented the DEX-mediated suppression of Bcl-2 (*P* < *0.05*) (Fig. 9b, c’’’). Meanwhile, the results of Annexin V-FITC/PI staining analysis manifested that SB obviously suppressed apoptosis of osteoblasts induced by DEX (*P* < *0.05*) (Fig. 9d–d’’’). Thus, we confirmed that SB had a substantial inhibition on apoptosis of osteoblasts derived from BMSCs via the caspase-3 pathway.

## Discussion

The suppression of bone formation and the promotion of bone destruction and apoptosis were confirmed to play pivotal roles in the process of SONFH (Yang et al. 2018). This current study revealed that HG exerted coordinated effects on the treatment of SONFH in rabbits, including an increase of osteocyte formation and a reduction of cell apoptosis in the femoral head tissue. In order to study the regulatory mechanism in greater detail, in vitro, we demonstrated that HG could enhance osteogenesis by stimulating BMSCs osteogenic differentiation and inhibit apoptosis in the osteoblast differentiated from BMSCs. Furthermore, combined with the UPLC analysis of HG, we found that NG displayed osteogenesis via stimulating BMSCs to osteoblasts, and SB inhibited the osteoblast apoptosis induced by excessive DEX, which confirmed the pharmacological activities in vivo and provided the evidence of active compounds in HG.

In this study, results from SEM and micro-CT demonstrated that long-term application of DEX markedly led to bone defect, trabecular fracture, cell apoptosis, and eventually progressed SONFH in rabbits (Fig. 2). Compared with the DEX induction, intra-articular injection of HG substantially decreased bone defects, increased bone formation and improved bone quality (Fig. 3a–e’’). Additionally, TUNEL-positive-stained cells in HG-treated rabbits exhibited a lower ratio (Fig. 3f–i), suggesting that HG remarkably attenuated the cell apoptosis induced by DEX. These data indicated that the intra-articular injection routine assured the HG exposure of femoral head tissue and the direct therapeutic effects on SONFH in rabbits and also suggested the underlying mechanisms contributing to this bone protection might rely on the stimulation of osteocytes formation or the inhibition of cell apoptosis.

To better understand the molecular mechanisms by which HG exerts bone protection against DEX injury, it is important to understand the molecular basis of DEX-induced bone loss. Among various hypotheses, the DEX-driven suppression of osteogenic differentiation and exacerbation of osteoblasts apoptosis were widely recognized (Tao et al. 2017; Liu et al. 2018). To validate whether HG functioned via these mechanisms, osteoblasts derived from BMSCs differentiation in vitro were conducted. Our data demonstrated that HG in vitro significantly contributed to osteogenic differentiation and a substantial increase in matrix mineralization compared with excessive DEX-treated BMSCs (Fig. 6f–g’’’), and the mRNA and protein expression of osteogenic differentiation-related markers, OCN and Runx2 (Huang et al. 2007), were also upregulated (Fig. 6a–e), indicating that HG had potential for osteoblast differentiation. However, the differentiation promoting effect did not stand to reason that HG inhibited the cell apoptosis in the femoral head tissue of SONFH. Osteoblasts differentiated from BMSCs were exposed to excessive DEX to induce apoptosis. We found that HG inhibited the osteoblast apoptosis assayed with Annexin V-FITC/PI staining (Fig. 8d–d’’’), meanwhile, upregulation of Bcl-2 and downregulation of Cyt c, Bax, PARP, and caspase-3 were observed (Elmore 2007; Xu et al. 2014; Feng et al. 2017) (Fig. 8a–c’’’’). In combination, these findings suggested a coordinated mechanism of action by which HG functioned at different stages of osteogenesis to mitigate DEX-induced bone loss.

Since the direct intra-articular injection of HG got access to the local tissue of the femur head, the components stimulating BMSCs osteogenic differentiation and anti-apoptosis of osteoblasts were unknown. Thus, UPLC analysis identified 42 compounds in HG (Fig. 4). By retrieving the published data, we found that NG and SB had been reported to be active substances in the treatment of SONFH (Huang et al. 2018; Ma et al. 2020). Consistent with the osteogenic effect of NG (Wang et al. 2015) in previous studies, our results suggested that NG in vitro increased the expression of β-catenin, OCN, and Runx2 (Fig. 7b–c’’’) to promote BMSCs differentiation to osteoblasts ALP expression and calcium mineralization (Fig. 7d–e’’’). In addition, the Annexin V-FITC/PI staining showed that SB protected osteoblasts derived from BMSCs against GCs-mediated apoptosis (Fig. 9d–d’’’) by increasing the ratio of Bcl-2/Bax and inhibiting the phosphorylation of caspase-3 (Fig. 9b–c’’’). Therefore, NG and SB were identified to be the active ingredients of HG, and the therapeutic effects and mechanism of actions were assumed to be representative, at least partly.

Our results indicated that the intra-articular injection of HG was effective in the treatment of SONFH by stimulating BMSCs osteogenic differentiation via the Wnt/β-catenin signaling pathway and inhibiting apoptosis of osteoblasts through decreasing caspase-3 and Bax expression and upregulating Bcl-2 expression. Additionally, future research may need to elucidate the specific role of other active ingredients in HG.

## Conclusion

In summary, our results suggested that the intra-articular injection of HG was effective in the treatment of SONFH by stimulating BMSCs osteogenic differentiation and inhibiting apoptosis of osteoblasts, by which we provided the experimental evidence that HG exerted the therapeutic effects in a multi-target mode. Although we identified two active components, further research needs to elucidate the roles of other ingredients in HG. Consequently, our study demonstrates that HG is a promising agent with therapeutic effects on SONFH patients.

### Supplementary Information

Below is the link to the electronic supplementary material.

Supplementary file1 (DOCX 14 KB)

Supplementary file2 (DOCX 13 KB)

Supplementary file3 (DOCX 26 KB)

## Data Availability

The raw data supporting the conclusions of this article will be made available by the authors, without undue reservation.

## Abbreviations

DEX: Dexamethasone
SONFH: Steroid-induced osteonecrosis of the femoral head
HG: Huogu injection
OCN: Osteocalcin
Runx2: Runt-related transcription factor 2
Cyt c: Cytochrome c
PARP: Poly [ADP-ribose] polymerase
NG: Naringin
SB: Salvianolic acid B
NONFH: Nontraumatic osteonecrosis of the femoral head
THA: Total hip arthroplasty
GCs: Glucocorticoids
BMSCs: Bone marrow mesenchymal stem cells
UPLC: Ultra-high performance liquid chromatography
MPS: Methylprednisolone
SEM: Scanning electron microscopy
ROI: Region of interest
BS/BV: Bone surface per bone volume
BS/TV: Bone surface per total volume
BV/TV: Bone volume fraction
BMD: Bone mineral density
Tb.N: Trabecular number
Tb.Th: Trabecular thickness
Tb.Sp: Trabecular separation
Conn.D: Connectivity density
Tt.Ar: Total bone area
Ct.Ar: Cortical area
Ct.Th: Cortical thickness
ALP: Alkaline phosphatase
OIM: Osteogenic induced medium
ARS: Alizarin red staining
qRT-PCR: Quantitative reverse transcription-polymerase chain reaction
