# Supplementary material for: Huogu injection protects against SONFH by promoting osteogenic differentiation of BMSCs and preventing osteoblast apoptosis
Source: Cell Tissue Res. 2023 Dec 2;395(1):63–79. doi: 10.1007/s00441-023-03846-7 (PMC10774174; doi:10.1007/s00441-023-03846-7)
Supplement: Supplementary file 1 — Supplementary file1 (DOCX 14 KB) [file 441_2023_3846_MOESM1_ESM.docx]

**Supplementary Data 1**

| **Antibody** | **Dilution ratio** | **Company** | **Molecular weight** | **Article No.** | **Nation** |
| --- | --- | --- | --- | --- | --- |
| PARP/cleaved-PARP antibody | 1：500 | wanleibio | 89, 116 kDa | WL01932 | China |
| caspase 3/cleaved caspase 3 antibody | 1：500 | wanleibio | 34, 20 kDa | WL02117 | China |
| Bax antibody | 1：500 | wanleibio | 21kDa | WL01637 | China |
| Bcl-2 antibody | 1：500 | wanleibio | 26 kDa | WL01556 | China |
| Cytochrome C antibody | 1：500 | wanleibio | 15 kDa | WL02410 | China |
| OCN antibody | 1：1000 | affinity | 11KDa | DF12303 | China |
| Runx2 antibody | 1：1000 | wanleibio | 57 KDa | WL03358 | China |
| β-catenin | 1：1000 | Servicebio | 92 KDa | GB11015 | China |
| β-actin antibody | 1：1000 | wanleibio | 42 kDa | WL01372 | China |
| goat anti rabbit IgG-HRP | 1：5000 | wanleibio | -- | WLA023 | China |
