# Supplementary material for: Huogu injection protects against SONFH by promoting osteogenic differentiation of BMSCs and preventing osteoblast apoptosis
Source: Cell Tissue Res. 2023 Dec 2;395(1):63–79. doi: 10.1007/s00441-023-03846-7 (PMC10774174; doi:10.1007/s00441-023-03846-7)
Supplement: Supplementary file 2 — Supplementary file2 (DOCX 13 KB) [file 441_2023_3846_MOESM2_ESM.docx]

**Supplementary Data 2**

| Gene | F（5'-3'） | R（5'-3'） |
| --- | --- | --- |
| Cytochrome C | TGGGCCAAATCTCCATGGTC | AGGCAGTGGCCAATTATTACTCA |
| PARP | CTGTTGGGAGAAGTTGCCCT | GAAATCCCGGTCCCAAGAGG |
| caspase-3 | GGAGCTTGGAACGCGAAGAA | ACACAAGCCCATTTCAGGGT |
| Bax | GCCTCCTTTCCTACTTCGGG | GGTTTATTGGCACCTCCCCC |
| Bcl-2 | GAGGGGCTACGAGTGGGATA | CGGTAGCGACGAGAGAAGTC |
| OCN | AAGTCCCACACAGCAACTCG | TCCATTGTTGAGGTAGCGCC |
| RUNX2 | TCGCCTCACAAACAACCACA | AGGCTGTTTGACGCCATAGT |
| β-catenin | ATCATTCTGGCCAGTGGTGG | GACAGCACCTTCAGCACTCT |
| GAPDH | TGCCACTCAGAAGACTGTGG | GGATGCAGGGATGATGTTCT |
