# Supplementary material for: Huogu injection protects against SONFH by promoting osteogenic differentiation of BMSCs and preventing osteoblast apoptosis
Source: Cell Tissue Res. 2023 Dec 2;395(1):63–79. doi: 10.1007/s00441-023-03846-7 (PMC10774174; doi:10.1007/s00441-023-03846-7)
Supplement: Supplementary file 3 — Supplementary file3 (DOCX 26 KB) [file 441_2023_3846_MOESM3_ESM.docx]

**Supplementary Data 3**

| No. | t_R_  (min) | Formula | Caculated mass (ESI^+^) | Measured mass (ESI^+^) | Mass error (ESI^+^) | Measured mass (ESI^-^) | Mass error (ESI^-^) | MS Fragment | | Identification | Structure | Source | Reference |
| --- | --- | --- | --- | --- | --- | --- | --- | --- | --- | --- | --- | --- | --- |
|  |  |  |  |  |  |  |  | ESI+ | ESI- |  |  |  |  |
| 1 | 0.73 | C_10_H_13_NO_2_ | 180.1025 | 180.1028 | 0.3 | 178.0864 | -0.4 | 166.0870, 163.0759, 149.0605, 145.0652 | 164.0704 | Phenylalanine methyl ester or isomer | Amino acids | N/A | N/A |
| 2 | 1.09 | C_9_H_11_NO_2_ | 166.0868 | 166.0874 | 0.6 | 164.0709 | -0.3 | 149.0612 | N/A | Phenylalanine | Amino acids | LC/SM | 1 |
| 3 | 1.60 | C_10_H_13_NO_2_ | 180.1025 | 180.1034 | 0.9 | 178.0875 | 0.7 | 163.0763, 145.0656 | N/A | Phenylalanine methyl ester or isomer | Amino acids | N/A | N/A |
| 4 | 2.71 | C_10_H_11_NO_2_ | 178.0868 | 178.0870 | 0.2 | 176.0722 | 1.0 | 162.0554 | 160.0378 | Methyl 2-amino-3-phenyl-2-propenoate | Amino acids | N/A | N/A |
| 5 | 3.84 | C_14_H_20_O_8_ | N/A | N/A | N/A | 315.1082 | 0.2 | N/A | 153.0553, 135.0454 | 3,4-Dihydroxyphenethyl glucoside | Phenolic acids | N/A | N/A |
| 6 | 4.01 | C_16_H_18_O_9_ | N/A | N/A | N/A | 353.0875 | 0.2 | N/A | 191.0542, 179.0328, 135.0443 | Neochlorogenic acid | Phenolic acids | LC | 1 |
| 7 | 5.08 | C_7_H_6_O_3_ | N/A | N/A | N/A | 137.0247 | -0.8 | N/A | 119.0100, 109.0280 | Protocate chualdehyde | Phenolic acids | LC | 1 |
| 8 | 6.27 | C_16_H_18_O_9_ | N/A | N/A | N/A | 353.0878 | 0.5 | N/A | 191.0565 | Chlorogenic acid | Phenolic acids | LC | 1 |
| 9 | 6.53 | C_12_H_13_NO_3_ | 220.0974 | 220.0973 | -0.1 | 218.0817 | 0.0 | 137.0607 | 188.0701, 162.0558, 135.0439 | (*Z*)-Methyl 2-acetamido-3-phenylacrylate | Amino acids | N/A | N/A |
| 10 | 6.66 | C_16_H_18_O_9_ | N/A | N/A | N/A | 353.0876 | 0.3 | N/A | 191.0564, 179.0347, 173.0459, 135.0450 | Crytochlorogenic acid | Phenolic acids | LC | 1 |
| 11 | 6.84 | C_9_H_8_O_4_ | N/A | N/A | N/A | 179.0344 | -0.4 | N/A | 135.0438, 107.0525 | Caffeic acid | Phenolic acids | SM/DR | 1 |
| 12 | 7.39 | C_16_H_18_O_10_ | 371.0978 | 371.0977 | -0.1 | 369.0830 | 0.8 | 209.0455, 192.0431, 163.0392 | 207.0295 | Fraxetin | Coumarins | LC | 5 |
| 13 | 7.51 | C_16_H_18_O_10_ | 371.0978 | 371.0981 | 0.3 | 369.0858 | 3.6 | 209.0468, 192.0408, 163.0396 | 207.0293 | Isofraxetin | Coumarins | LC | 5 |
| 14 | 8.48 | C_25_H_24_O_12_ | N/A | N/A | N/A | 515.1190 | 0.0 | N/A | 353.0868, 191.0569, 179.0341, 135.0457 | 3,5-O-dicaffeoyl-quinic acid isomer | Phenolic acids | LC | 1 |
| 15 | 9.45 | C_12_H_16_O_4_ | 225.1127 | 225.1122 | -0.5 | N/A | N/A | 207.1021, 189.0918, 179.1087, 165.0924, 137.0974 | N/A | Senkyunolide I/H | Phthalides | LC | 1 |
| 16 | 10.04 | C_12_H_18_O_4_ | 225.1127 | 225.1124 | -0.3 | N/A | N/A | 207.1025, 165.0911, 137.0969 | N/A | Senkyunolide I/H | Phthalides | LC | 1 |
| 17 | 10.71 | C_15_H_12_O_6_ | N/A | N/A | N/A | 287.0573 | 1.7 | N/A | 179.0351, 161.0262 | Eriodictyol | Flavonoids | DR | 4 |
| 18 | 11.00 | C_12_H_16_O_4_ | 227.1283 | 227.1288 | 0.5 | N/A | N/A | 209.1177, 191.1071, 163.1129 | N/A | Senkyunolide D | Phthalides | LC | 1 |
| 19 | 11.27 | C_10_H_8_O_4_ | 193.0501 | 193.0504 | 0.3 | N/A | N/A | 175.0391, 147.0455, 137.0613 | N/A | Scopoletin | Coumarins | LC | 1 |
| 20 | 11.36 | C_10_H_8_O_4_ | 193.0501 | 193.0499 | -0.2 | N/A | N/A | 147.0418, 137.0598 | N/A | Scopoletin and isomer | Coumarins | LC | 1 |
| 21 | 11.41 | C_25_H_24_O_12_ | N/A | N/A | N/A | 515.1194 | 0.4 | N/A | 353.0878, 191.0354, 179.0344, 173.0459, 135.0446 | 3,4-O-dicaffeoyl-quinic acid | Phenolic acids | N/A | N/A |
| 22 | 11.69 | C_25_H_24_O_12_ | N/A | N/A | N/A | 515.1191 | 0.1 | N/A | 353.0867, 191.0552, 179.0339, 135.0450 | 3,5-O-dicaffeoyl-quinic acid | Phenolic acids | LC | 1 |
| 23 | 11.80 | C_27_H_32_O_14_ | N/A | N/A | N/A | 579.1679 | -3.5 | N/A | 271.0583 | Naringin | Flavonoids | DR | 4 |
| 24 | 12.19 | C_36_H_30_O_16_ | N/A | N/A | N/A | 717.1461 | 0.5 | N/A | 519.0933, 339.0502, 321.0399, 295.0609, 277.0489, 185.0248 | Salvianolic acid B and isomers | Phenolic acids | SM | 3 |
| 25 | 12.25 | C_25_H_24_O_12_ | N/A | N/A | N/A | 515.1183 | -0.7 | N/A | 353.0868, 191.0554, 179.0336, 173.0452, 135.0442 | 4,5-O-dicaffeoyl-quinic acid | Phenolic acids | N/A | N/A |
| 26 | 12.44 | C_18_H_16_O_8_ | N/A | N/A | N/A | 359.0775 | -0.8 | N/A | 197.0456, 179.0345, 161.0243, 135.0452 | Rosmainic acid | Phenolic acids | LC/SM | 1,3 |
| 27 | 12.46 | C_12_H_14_O_3_ | 207.1021 | 207.1023 | 0.2 | N/A | N/A | 189.0917, 179.1074, 161.0971 | N/A | Senkyunolide F | Phthalides | LC | 1 |
| 28 | 12.52 | C_27_H_22_O_12_ | 539.1190 | 539.1180 | -1.0 | 537.1033 | 1.6 | 521.1072, 341.0663 | 493.1148, 313.0715, 295.0613, 203.0353, 185.0242, 159.0454, 135.0457 | Salvianolic acid H/I/J/isomer | Phenolic acids | SM | 3 |
| 29 | 12.63 | C_27_H_22_O_12_ | 539.1190 | 539.1194 | 0.4 | 537.1033 | -0.3 | 521.1086, 341.0666 | 493.1139, 313.0725, 295.0616, 203.0352, 185.0248, 159.0448, 135.0449, 109.0304 | Salvianolic acid H/I/J/isomer | Phenolic acids | SM | 3 |
| 30 | 13.04 | C_12_H_14_O_3_ | 207.1021 | 207.1026 | 0.5 | N/A | N/A | 189.0901, 179.1059, 161.0242 | N/A | Senkyunolide F Isomers | Phthalides | LC | 1 |
| 31 | 13.21 | C_36_H_30_O_16_ | 719.1612 | 719.1608 | -0.4 | 717.1455 | -0.1 | 701.1494, 521.1078, 323.0555 | 519.0924, 339.0497, 321.0396, 295.0600, 277.0499, 185.0237 | Salvianolic acid B | Phenolic acids | SM | 1,3 |
| 32 | 13.34 | C_26_H_22_O_10_ | N/A | N/A | N/A | 493.1136 | 0.1 | N/A | 313.0708, 295.0607, 203.0347, 185.0241, 159.0455, 135.0448, 109.0293 | Salvianolic acid A isomer | Phenolic acids | SM | 3 |
| 33 | 13.48 | C_36_H_30_O_16_ | N/A | N/A | N/A | 717.1454 | -0.2 | N/A | 519.0923, 339.0505, 321.0407, 295.0598 | Salvianolic acid B and isomers | Phenolic acids | SM | 3 |
| 34 | 13.59 | C_36_H_30_O_16_ | 719.1612 | 719.1596 | -1.6 | 717.1455 | -0.1 | 521.1093, 323.0551 | 519.0933, 339.0503, 321.0403, 295.0610 | Salvianolic acid B and isomers | Phenolic acids | SM | 3 |
| 35 | 13.71 | C_26_H_22_O_10_ | 495.1291 | 495.1299 | 0.8 | 493.1134 | 0.1 | 477.1183, 339.0883, 297.0768 | 313.0716, 295.0617, 203.0349, 185.0246, 159.0449, 135.0446, 109.0292 | Salvianolic acid A | Phenolic acids | SM | 1,3 |
| 36 | 14.10 | C_26_H_20_O_10_ | N/A | N/A | N/A | 491.0982 | 0.4 | N/A | 339.0504, 321.0396, 311.0566, 293.0443 | Salvianolic acid C | Phenolic acids | SM | 1 |
| 37 | 15.39 | C_12_H_14_O_3_ | 207.1021 | 207.1035 | 1.4 | 205.0870 | 0.5 | 189.0919, 171.0814 | 161.0973 | Senkyunolide F Isomers | Phthalides | LC | 1 |
| 38 | 15.48 | C_12_H_14_O_2_ | 191.1078 | 191.1072 | -0.6 | N/A | N/A | 163.1125, 149.0612, 135.0450, 79.0559 | N/A | Z-Liqustilide | Phthalides | LC | 2 |
| 39 | 15.50 | C_15_H_10_O_7_ | N/A | N/A | N/A | 301.0338 | -1.0 | N/A | N/A | Quercetin | Flavonoids | DR | 4 |
| 40 | 16.07 | C_16_H_22_O_4_ | 279.1596 | 279.1599 | 0.3 | N/A | N/A | 261.1484, 233.1545 | N/A | Senkyunolide M/Q | Phthalides | LC | 1 |
| 41 | 16.34 | C_16_H_22_O_4_ | 279.1596 | 279.1595 | -0.1 | N/A | N/A | 261.1492, 233.1545 | N/A | Senkyunolide M/Q | Phthalides | LC | 1 |
| 42 | 16.80 | C_19_H_22_O_4_ | 337.1429 | 337.1405 | -2.4 | 313.1439 | -0.1 | 297.1491, 253.1595 | 269.1525, 226.0969, 213.1269 | Neocryptotanshinone | Diterpenoid quinones | SM | 3 |

Salvia Miltiorrhiza: SM; Ligusticum Chuanxiong: LC; Drynariae Rhizoma: DR

N/A: Not applicable

1. Chen, X.; Lou, Z.; Zhang, H.; Tan, G.; Liu, Z.; Li, W.; Zhu, Z.; Chai, Y., Identification of multiple components in Guanxinning injection using hydrophilic interaction liquid chromatography/time-of-flight mass spectrometry and reversed-phase liquid chromatography/time-of-flight mass spectrometry. *Rapid communications in mass spectrometry: RCM* **2011,** *25*, 1661-74.
2. Guo, L.; Gong, M.; Wu, S.; Qiu, F.; Ma, L., Identification and quantification of the quality markers and anti-migraine active components in Chuanxiong Rhizoma and Cyperi Rhizoma herbal pair based on chemometric analysis between chemical constituents and pharmacological effects. *Journal of ethnopharmacology* **2020,** *246*, 112228.
3. Yang, S. T.; Wu, X.; Rui, W.; Guo, J.; Feng, Y. F., UPLC/Q-TOF-MS Analysis for Identification of Hydrophilic Phenolics and Lipophilic Diterpenoids from Radix Salviae Miltiorrhizae. *Acta Chromatographica* **2015,** *27*, 711-728.
4. Xu, Z. L.; Xu, M. Y.; Wang, H. T.; Xu, Q. X.; Liu, M. Y.; Jia, C. P.; Geng, F.; Zhang, N., Pharmacokinetics of Eight Flavonoids in Rats Assayed by UPLC-MS/MS after Oral Administration of Drynariae rhizoma Extract. *Journal of analytical methods in chemistry* **2018,** *2018*, 4789196
5. Yuan, X.; Han, B.; Feng, Z. M.; Jiang, J. S.; Yang, Y. N.; Zhang, P. C., Chemical constituents of Ligusticum chuanxiong and their anti-inflammation and hepatoprotective activities. *Bioorganic chemistry* **2020,** *101*, 104016.

### Chemical Composition Analysis of Huogu injection

We evaluated the chemical composition of Huogu injection to determine how it exerts its therapeutic effects on SONFH using positive and negative ion mode UPLC-Q-TOF-MS. Total ion chromatograms (TIC) of Huogu injection are presented in Fig.1. Through analysis, 42 compounds of Huogu injection had been identified and its information of retention time, compound name, formula, error and its source were presented at Table1. The 42 Huogu injection compounds identified included 5 amino acids, 20 phenolic acids, 4 coumarins, 9 phthalides, 3 flavonoids, and 1 diterpenoid quinones, most which were derived from the herbs Danshen (13 compounds), Chuanxiong (20 compounds), and Gusuibu (4 compounds).
